# Integrative satellitomics reveals distinct patterns of organization, transcription and evolution of satellite DNAs in *Tenebrio molitor*

**DOI:** 10.1186/s13062-026-00808-1

**Published:** 2026-05-08

**Authors:** Patrik Majcen, Antonio Sermek, Đurđica Ugarković, Brenda Oppert, Miroslav Plohl, Eva Šatović-Vukšić

**Affiliations:** 1 Ruđer Bošković Institute, Bijenička 54, Zagreb, 10000 Croatia; 2 Kansas State University, Manhattan, 66502 KS USA; 3 Entovations LLC, Manhattan, 66503 KS USA

**Keywords:** Satellite DNA, Satellitome, Extrachromosomal circular DNA, Transcription, Phylogeny, Molecular evolution, Insect genomics, *Tenebrio molitor*

## Abstract

Satellite DNAs (satDNAs) are repetitive sequences that play important roles in chromosomal architecture, genome evolution, and regulation. Here, we present a comprehensive characterization of *Tenebrio molitor* satellitome, integrating cytogenetic mapping, in silico genome annotation, divergence profiling, screening of extrachromosomal circular DNA (eccDNA), transcription analysis across developmental stages, and phylogenetic and age analyses. SatDNAs exhibited diverse chromosomal organizations, ranging from widespread to chromosome-restricted distributions. Discrepancies between assembly-based and physical mapping highlight limitations of individual approaches and underscore the importance of their integration. Divergence landscape analyses revealed different homogenization efficiencies and turnover rates, reflecting distinct evolutionary trajectories among individual satDNAs. Phylogenetic reconstruction revealed distinct patterns which include clear species-specific clustering of monomers, mixed interspecific clustering, and dispersed topologies. Comparative analyses across insect orders enabled age estimation, identifying both ancient (≥380 MYA) and lineage-specific satDNAs, apparently restricted to *T. molitor*. We designed and applied an approach that enables the simultaneous detection of multiple satDNAs within the eccDNA fraction which confirmed the presence of six satDNAs in eccDNA. RNA-seq analyses revealed coordinated, stage-specific transcription of all satDNAs, with elevated expression in late male pupae and early male adults. Together, these results reveal a highly dynamic, heterogeneous, and functionally relevant satDNA landscape in *T. molitor* and demonstrate the importance of integrative approaches for understanding molecular mechanisms and trajectories of satDNA evolution.

**Supplementary Information:**

The online version contains supplementary material available at 10.1186/s13062-026-00808-1.

## Introduction

Although sometimes referred to as the “dark matter of the genome”, repetitive sequences are recognized as key components of genome structure and as drivers of its evolution, since genome dynamics is strongly influenced by processes that reorganize repetitive DNAs, and modify their composition and copy number [1–5]. Satellite DNAs (satDNAs) are sequences repeated in tandem, frequently located in (peri)centromeric and subtelomeric heterochromatin, forming arrays of hundreds to thousands of monomers [3, 6–8]. They can also be found throughout euchromatin, frequently near genes or within introns, occurring as individual repeats or short arrays [9]. SatDNAs contribute to genome evolution through their involvement in speciation processes and play an essential role in maintaining the genome within the nucleus (reviewed in [10]). Increasing evidence also points to active and tightly regulated satDNA transcription, with satDNA transcripts participating in diverse cellular processes. For instance, satDNA transcripts are implicated in heterochromatin establishment, centromere function, and gene expression regulation across various biological contexts, stress responses, and environmental adaptations (reviewed in [11]). Moreover, aberrant satDNA transcription has been linked to carcinogenesis, and satDNA transcripts show potential as biomarkers for cancer detection [11]. SatDNA transcription is often developmentally regulated and tissue-specific, indicating functional relevance [12, 13]. Mechanisms that are proposed to contribute to the dispersal of satDNA sequences throughout the genome are unequal crossing over, transposition and extrachromosomal circular DNA (eccDNA) formation and reintegration [8, 14]. EccDNA molecules are circular DNA elements derived from chromosomal sequences and are recognized as widespread components of eukaryotic genomes. Tandemly repeated sequences, including satDNAs, are particularly prone to eccDNA formation [15], due to their organization and susceptibility to intra-chromosomal homologous recombination and excision of circular forms. It is suggested that eccDNAs may contribute to satDNA array length changes and satDNA homogenization, as well as genomic plasticity [16].

The yellow mealworm, *Tenebrio molitor,* is becoming a model organism of interest in a number of studies in biology, biochemistry, evolution, immunology and physiology [17–24]. Extracts from *T. molitor* have demonstrated antiproliferative activity against colorectal adenocarcinoma and hepatocellular carcinoma [25], as well as wound-healing and antimicrobial properties [26, 27]. Furthermore, this species produces antifreeze proteins that can be used for vegetable preservation [28]. Due to its high protein content and nutritional value, *T. molitor* is increasingly used as an animal dietary supplement and has been introduced to the human food market [29–31]. The importance of studying *T. molitor* is highlighted by ongoing efforts to improve the genome assembly and annotation [32–34]. While repetitive regions pose significant technical challenges for DNA sequencing and assembly [35], a substantial portion of the *T. molitor* genome consists of a single satDNA. This satDNA has a monomer length of 142 bp and is localized in the pericentromeric heterochromatin of all chromosomes, as confirmed by in situ restriction digestion of metaphase chromosomes [36–38]. The most recent, improved genome assembly of *T. molitor* was accompanied by in silico satellitome analysis [34], in which the abundance of the major 142-bp satDNA was determined to be 26.5% (TmSat01). Ten additional low-abundant satDNAs were identified (TmSat02 - TmSat11), together constituting 1.5% of the genome assembly [34]. However, the exact chromosomal localization and distribution patterns of *T. molitor* satDNAs remained unknown, as well as their transcription and divergence profiles. As genomic databases continue to expand with an exponentially increasing number of sequenced genomes, newly available data allow for more comprehensive and accurate insights into satDNA distribution across taxa, including the profiling of satDNA conservation and divergence, and a more detailed understanding of the relationships between satDNA distribution patterns and species phylogeny.

In this study, we determined the localization of all satDNAs (TmSat01–11) in the genome of *T. molitor* using complementary in situ and in silico approaches. We analyzed their transcription across different developmental stages in males and females using RNA-seq data. Furthermore, we designed and applied an approach that enables the simultaneous detection of multiple satDNAs within the eccDNA fraction, confirming the presence of six satDNAs in eccDNA. We additionally examined sequence divergence for each *T. molitor* satDNA and assessed their presence across other insect species. These analyses allowed us to reconstruct satDNA phylogenies, explore relationships between satDNA distribution and species phylogeny, and estimate the minimal age of each satDNA family.

This study represents the first comprehensive analysis of a species’ satellitome, integrating simultaneously satDNA genomic organization, inter- and intra-species distribution, evolutionary dynamics, eccDNA association and transcriptional activity. By combining cytogenetic, genomic, phylogenetic and transcriptomic data, we provide a wider perspective of satDNA dynamics across structural, functional, taxonomical and temporal levels. Our results underline the importance of such an integrative framework for understanding the complexity of satDNA biology and support its broader application in future studies of repetitive DNA in other species.

## Materials and methods

### SatDNAs annotation, divergence profiling, and genomic context inspection

Consensus sequences of the 11 *T. molitor* satDNAs (NCBI accession numbers PX395431–PX395441) were annotated on two recent publicly available chromosome-level genome assemblies of this species, GCA_037075425.1 and GCA_963966145.1. Monomer consensus sequences were used to annotate each satDNA on chromosomes and scaffolds in Geneious Prime software (Biomatters Ltd., Auckland, New Zealand), allowing up to 45% divergence from the consensus to detect monomer variants. To assess sequence divergence, all monomers of each satDNA were extracted from the genome and compared to their respective consensus sequences within Geneious Prime. Divergence landscapes were generated using Kimura 2-parameter distances and grouped into 1% divergence bins. Peaks were defined as local maxima in the abundance distribution, where a given bin or a series of neighboring bins exhibited 50% higher values than preceding and following bins. For the analysis of the surrounding regions of TmSat06 and TmSat07, the corresponding arrays, along with 1,000 bp of flanking sequences upstream and downstream of each array, were extracted and analyzed in Geneious Prime. All nucleotide sequence alignments were performed in the same software.

The TideCluster software tool, implemented within the RepeatExplorer Galaxy platform (https://repeatexplorer-elixir.cerit-sc.cz/galaxy/), was used for *de novo* detection of tandem repeats in the reference genome assembly GCA_963966145.1. The TideCluster software integrates TideHunter [39] and TAREAN analyses [40], and was run using default parameters.

### Preparation of chromosome spreads from male pupae

The sex of the pupae was determined following Bhattacharya et al. [41]. Chromosome preparations were obtained from male pupae. Testes were dissected and immersed in distilled water for 45 min to induce osmotic shock. The tissue was then fixed in Carnoy’s solution (absolute ethanol:glacial acetic acid, 3:1) and subsequently macerated in 50% glacial acetic acid in an Eppendorf tube. Droplets of the suspension were spread onto glass slides and placed on a hot plate at 42 °C. After drying, the chromosomal preparations were dehydrated in series of ethanol solutions (70%, 90%, 100%; 30 s each). The slides were stored at −20 °C until utilized.

### Probe labelling

DNA probes for fluorescent in situ hybridization (FISH) were labelled by PCR. Each of the 20 µL reaction contained ~20 ng of DNA, 2.5 U GoTaq Flexi G2 DNA polymerase (Promega), GoTaq Buffer, 1.5 mM MgCl_2_, primers (0.1 µM each), 100 µM dATP/dCTP/dGTP (NEB), 50 µM dTTP (NEB), 50 µM biotin-16-dUTP (Jena Bioscience). After verification by agarose gel electrophoresis, DNA probes were purified using either the QIAquick PCR Purification Kit or the QIAquick Gel Extraction Kit (Qiagen). Probe concentrations were measured with a DeNovix fluorometer. Nucleotide sequences of the primers and PCR amplification conditions are provided in Supplementary Table S1.

### Fluorescent in situ hybridization (FISH)

FISH experiments were performed following the protocol outlined in Pérez-García et al. [42], with a modification in the pepsin digestion step, which was carried out for 5 min at 37 °C. Each FISH assay utilized 50 ng of satDNA probe. Prior to use, probes were denatured at 80 °C for 8 min and immediately cooled on ice for 2 min. Chromosomal DNA was denatured for 1 minute and 45 seconds in 50% formamide (Sigma-Aldrich) in 2× SSC. Signal detection was carried out using fluorescein-conjugated streptavidin (Vector Laboratories) at a 1:200 dilution, biotinylated anti-streptavidin (Vector Laboratories) at 1:100, followed by a second layer of fluorescein-conjugated streptavidin at 1:200. Finally, chromosomes were counterstained with 100 ng/mL DAPI (Sigma-Aldrich) and mounted in Mowiol 4–88 mounting medium (Sigma-Aldrich). Signals were visualized and captured using a Leica TCS SP8 X laser-scanning confocal microscope.

### Analyses of transcriptomes

Publicly available RNA-seq data from *T. molitor* was used for the analyses (NCBI BioProject accession PRJNA1012330). The dataset contains male and female samples spanning multiple developmental stages: eggs, early, mid, and late larvae, early and late pupae, and early and late adults. The number of accessible biological replicates varied between stages. Raw sequencing reads were evaluated for base quality and adapter content using FastQC (https://www.bioinformatics.babraham.ac.uk/projects/fastqc/). Adapter trimming and removal of low-quality bases were performed with TrimGalore (https://www.bioinformatics.babraham.ac.uk/projects/trim_galore/). Filtered paired-end reads were mapped to the *T. molitor* reference genome (RefSeq accession GCF_963966145.1, NCBI) using HISAT2 [43] with default parameters. Gene-level read counts were obtained with featureCounts [44]. To identify potentially problematic libraries, read count data were variance-stabilized using the rlog transformation (DESeq2 [45]), and subjected to principal component analysis (PCA) in R (R Core Team, 2024) (Supplementary Figure S1). Sample outliers were detected using a robust covariance estimator (EllipticEnvelope [46]); based on the first principal components. Libraries with extremely low sequencing depth or detected as outliers were excluded from subsequent analyses.

We constructed a custom database comprising 11 *T. molitor* satDNAs. Reads were mapped to satellite dimer sequences from this database to improve alignment specificity. Mapping was performed using Bowtie2 [47] with the -a option to report all alignments, allowing for a single mismatch. Mapped reads were counted using SAMtools [48]. Counts for each satDNA were normalized using the fragments per kilobase per million mapped reads (FPKM) method [49]. Due to significant copy number variation among satellites FPKM values are valid for longitudinal analysis (the same satellite DNA across different developmental stages) and not for comparison between different satellite DNAs. Data processing and visualization were carried out in R (https://www.R-project.org/) using the ggplot2 package [50]. Stage-specific colors and plotting aesthetics were customized to facilitate comparison across developmental stages.

### Extrachromosomal circular DNA (eccDNA) experiments

Genomic DNA was isolated from larvae using a standard phenol/chloroform protocol, followed by eccDNA isolation. To remove linear DNA fragments, 400 ng of purified DNA was digested with Exonuclease V (NEB). The initial digestion was performed in a 50 µL reaction containing 20 U of Exonuclease V, 5 µL of 10× NEBuffer 4, 5 µL of 10 mM ATP, and nuclease-free water to a final volume of 50 µL. The reaction was incubated at 37 °C for 15 hr. Subsequently, 5 µL ATP, 1 µL 10× NEBuffer 4, and 20 U Exonuclease V were added, and the reaction continued for additional 7 h at 37 °C. Further additions of 5 µL ATP, 0.1 µL 10× NEBuffer 4, and 20 U Exonuclease V were made, and the digestion proceeded for additional 18 h at 37 °C. Finally, 5 µL ATP, 0.1 µL 10× NEBuffer 4, and 30 U Exonuclease V were added, and digestion proceeded for 6 h at 37 °C. Afterwards, the reaction was heat-inactivated at 70 °C for 30 min.

To confirm the elimination of chromosomal linear DNA and the preservation of circular DNA, PCR was performed on Exonuclease V–treated sample. As a negative control for chromosomal DNA removal, PCR with primers targeting the histone H3 gene (TM_H3F 5′-CGCTTACCGTTCGAAG-3′ and TM_H3R 5′-TATTTGCAATTCGTCGTAG-3′) was performed. As a positive control for the presence of circular DNA, PCR with primers for the mitochondrial 16S gene (Tm16S_F 5′-GTATTTTGACTGTGCAAAG-3′ and Tm16S_R 5′-TTTTAATCCAACATCGAGG-3′) was performed. Primer sequences were designed based on *T. molitor* H3 gene (XM_069038316.1) and mitochondrial 16S sequences (EU048292, AJ633673.1, AJ438153.1), available in the NCBI database. PCR reactions (10 µL total volume) contained 5 µL 2× PCR Master Mix (Promega), 0.4 µL of each primer (10 pmol/µL), and 1 µL of the Exonuclease V–treated DNA. Amplification was carried out under the following conditions: initial denaturation at 95 °C for 5 min, followed by 25 cycles of 95 °C for 30 s, 50 °C for 30 s, and 72 °C for 30 s, with a final extension at 72 °C for 7 min.

### Phylogeny and age reconstruction

In order to perform phylogeny reconstruction, for each satDNA of *T. molitor*, 100 monomers were randomly subsampled from the genomic assembly GCF_963966145.1 in Geneious Prime. For other insect species, homologous satDNA sequences were retrieved by querying GenBank using the consensus sequences of TmSat01–TmSat11. Searches were performed with the blastn algorithm against both the Whole Genome Shotgun (WGS) and Nucleotide (nt) databases (GenBank + EMBL + DDBJ + PDB + RefSeq). All contigs with hits were downloaded, locally annotated with TmSat01-11 consensus sequences in Geneious Prime. Monomers were extracted and manually inspected. Where more than 100 were available, they were randomly subsampled to 100 sequences, following the same procedure applied to the *T. molitor* ones.

Multiple sequence alignments of satDNA monomers were performed using MAFFT alignment algorithm implemented in Geneious Prime. Maximum likelihood phylogenies were inferred using PhyML under the TN93 substitution model. Consensus trees were reconstructed using the Consensus Tree builder algorithm, and nodal support values were obtained through 100 bootstrap replicates. Divergence times of insect taxa were obtained via Timetree of life (TTOL) database [51]. Divergence time estimates for all taxa in the TTOL are pre-computed and stored in the TTOL database. The evolutionary timeline shown was constructed by the TTOL algorithm, by locating queried taxa within its precomputed database and traversing the tree toward the root, collecting divergence times at each internal node. The approximate evolutionary age of each satDNAs was inferred manually, based on its distribution across insect taxa, considering only species in which the query coverage exceeded 50% of the monomer sequence.

## Results

### In silico analysis of chromosomal distribution of *T. molitor* satDNAs

The chromosomal distribution of the 11 *T. molitor* satDNAs (TmSat01–TmSat11) was first examined in silico. For that purpose, we have annotated consensus sequences of each satDNA on the two recent chromosome-level assemblies of this species. The assembly GCA_037075425.1 [34] comprises linkage groups LG1–LG10 plus LGX and 1,006 unplaced scaffolds, while the assembly GCF_963966145.1 contains nine autosomes, the X and Y chromosomes, and 225 additional scaffolds. For both assemblies, the presence or absence of each of the 11 satDNAs was recorded for each chromosome or linkage group, along with the number of monomers on unplaced scaffolds (Table 1). A substantial difference in the number of annotated satDNA monomers was observed between the two assemblies. For example, TmSat01 is represented by only 1,689 monomers in GCF_963966145.1, compared to 12,407 monomers in GCA_037075425.1. Conversely, the low-abundance satDNAs TmSat02–TmSat11 are considerably more enriched in the GCF_963966145.1 assembly (Table 1). Therefore, the GCF_963966145.1 assembly, currently designated as the reference genome assembly, was used for subsequent analyses.

**Table 1:**
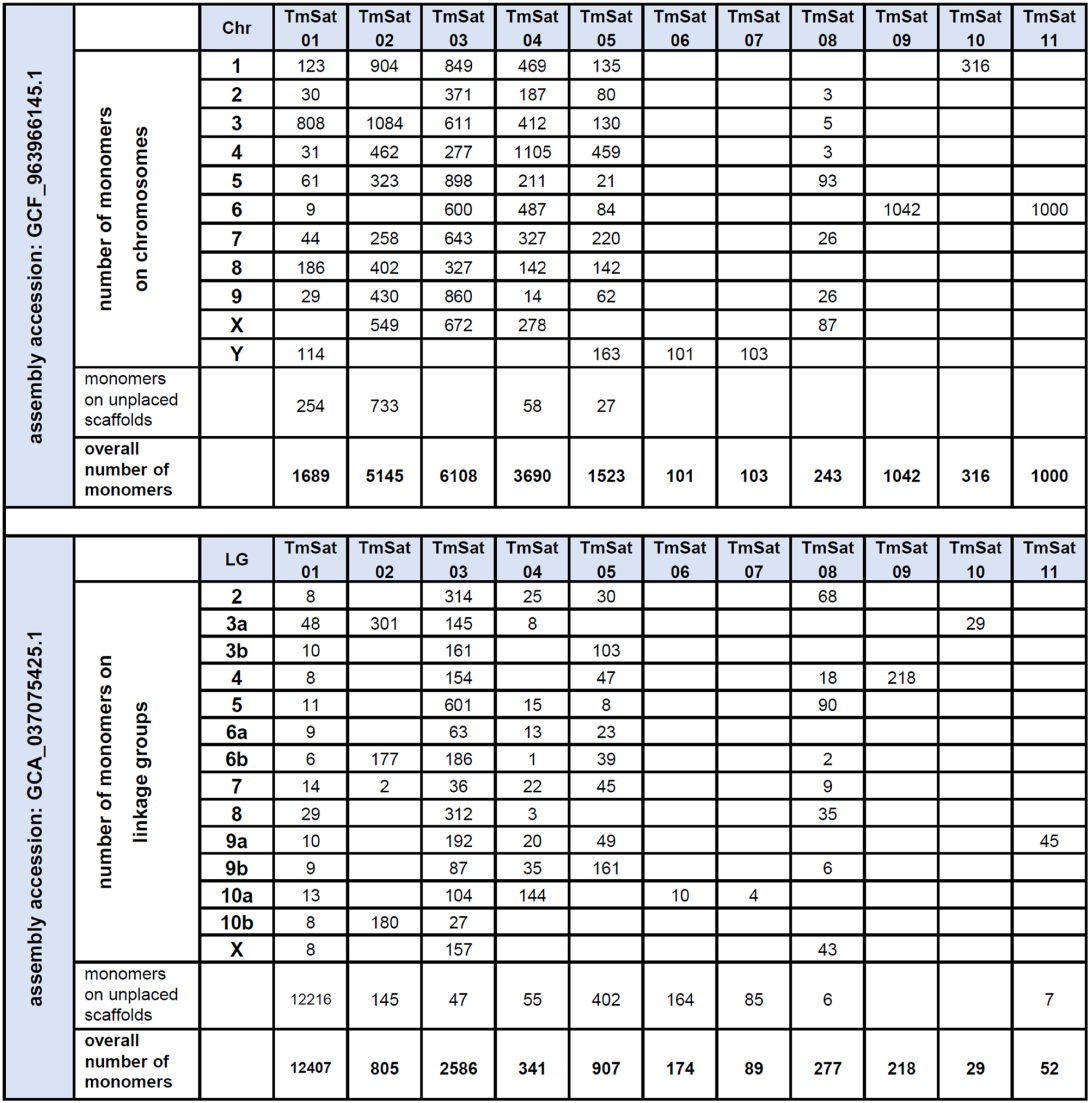
Abundance and distribution of monomers belonging to TmSat01–TmSat11 across two *T. molitor* genome assemblies

**Fig. 1:**
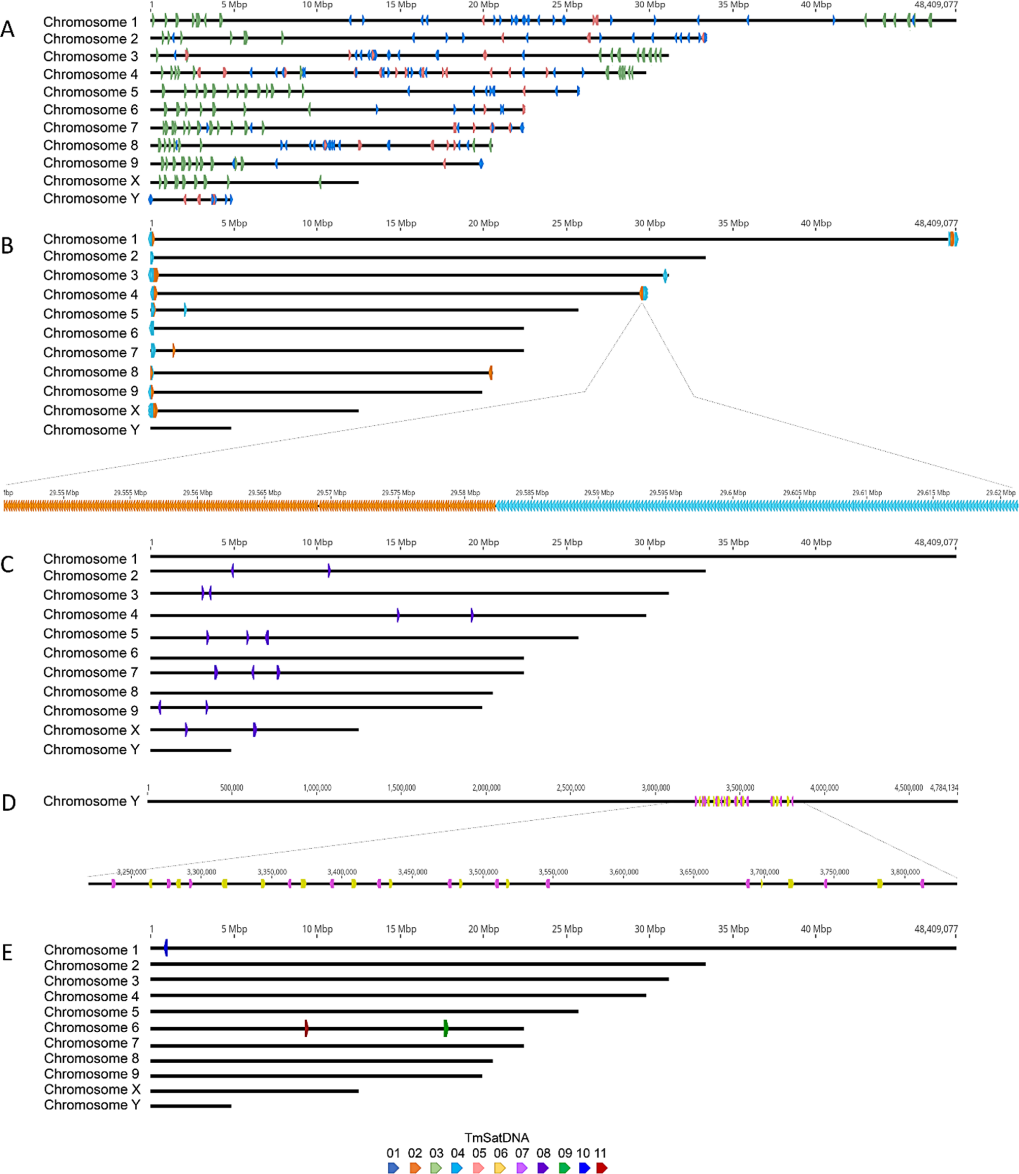
In silico chromosomal localization of TmSat01 - TmSat11 on the *T. molitor* genome assembly GCF_963966145.1. Monomers of each satDNA are represented by a distinct color, denoted in the legend. Panels correspond to: **A**) TmSat01, TmSat03, and TmSat05. **B**) TmSat02 and TmSat04. **C**) TmSat08. **D**) TmSat06 and TmSat07. **E**) TmSat09, TmSat10, and TmSat11

The eleven *T. molitor* satDNAs display distinct patterns of placement, distribution, and organization of monomer arrays on the chromosomes (Fig. 1). TmSat01, TmSat03, and TmSat05 occur as numerous interspersed arrays across the majority of chromosomes (Fig. 1A). TmSat02 and TmSat04 form several large terminal blocks on most chromosomes, often arranged in a way that the arrays of one satDNA directly follow those of the other (Fig. 1B). TmSat08 is organized into several blocks of arrays distributed across seven chromosomes (Fig. 1C). TmSat06 and TmSat07 are confined to the Y chromosome, where their arrays intertwine and are separated by blocks of unrelated sequences (Fig. 1D). Finally, TmSat09, TmSat10, and TmSat11 each form a single large block of consecutive monomers, TmSat09 and TmSat11 on chromosome 6, and TmSat10 on chromosome 1 (Fig. 1E).

The TmSat06 and TmSat07 were each detected in 12 short arrays (Fig. 1D), ranging from 2 to 9 monomers for TmSat06 and 6–11 for TmSat07. Given their organization in a small number of short arrays, we further explored their genomic context by analyzing the sequences flanking the arrays of both satDNAs. For TmSat06, segments preceding all arrays exhibited very high nucleotide sequence similarity (>99%), particularly within the 1,013 bp region directly prior to the arrays. Upstream from this segment, similarity persisted but was interrupted by blocks of insertions or deletions shared among subsets of sequences (Supplementary Figure S2A). Similarly, the regions following TmSat06 arrays showed conserved sequence similarity (>95%) within a 3,370 bp stretch directly downstream of the arrays (Supplementary Figure S2B). The exceptions are 3 extractions: one containing a 430 bp insertion, one showing similarity only over the first 1,000 bp, and one omitted due to a sequencing gap. In the case of TmSat07, the 2,890 bp region preceding the arrays retains sequence similarity across most sequences, interrupted by several indels up to 100 bp in size. Two sequences deviated from this pattern, one due to the insertion of a TmSat05 array (Supplementary Figure S3). Downstream of TmSat07 arrays, a 4,755 bp segment remained conserved, with indel blocks of up to 300 bp. The flanking sequences of TmSat06 and TmSat07 show no mutual similarity and lack detectable homology to any sequences in the NCBI or RepBase databases.

In addition to the TAREAN-obtained consensus-based annotation of the 11 satDNAs, *de novo* detection from the assembly was performed using TideHunter, allowing a comparison between assembly-free and assembly-based approaches for satDNA identification. Using TideHunter, seven satDNAs (TmSat01, TmSat02, TmSat03, TmSat04, TmSat05, TmSat08, and TmSat11) were identified within the main tandem repeat clusters (TRCs) (Supplementary Figure S4A). Some sequences belonging to TmSat08, as well as TmSat09 and TmSat10, were detected in additional clusters (Supplementary Figure S4B), whereas TmSat06 and TmSat07 were not detected by this approach.

### In situ localization of *T. molitor* satDNAs

**Fig. 2:**
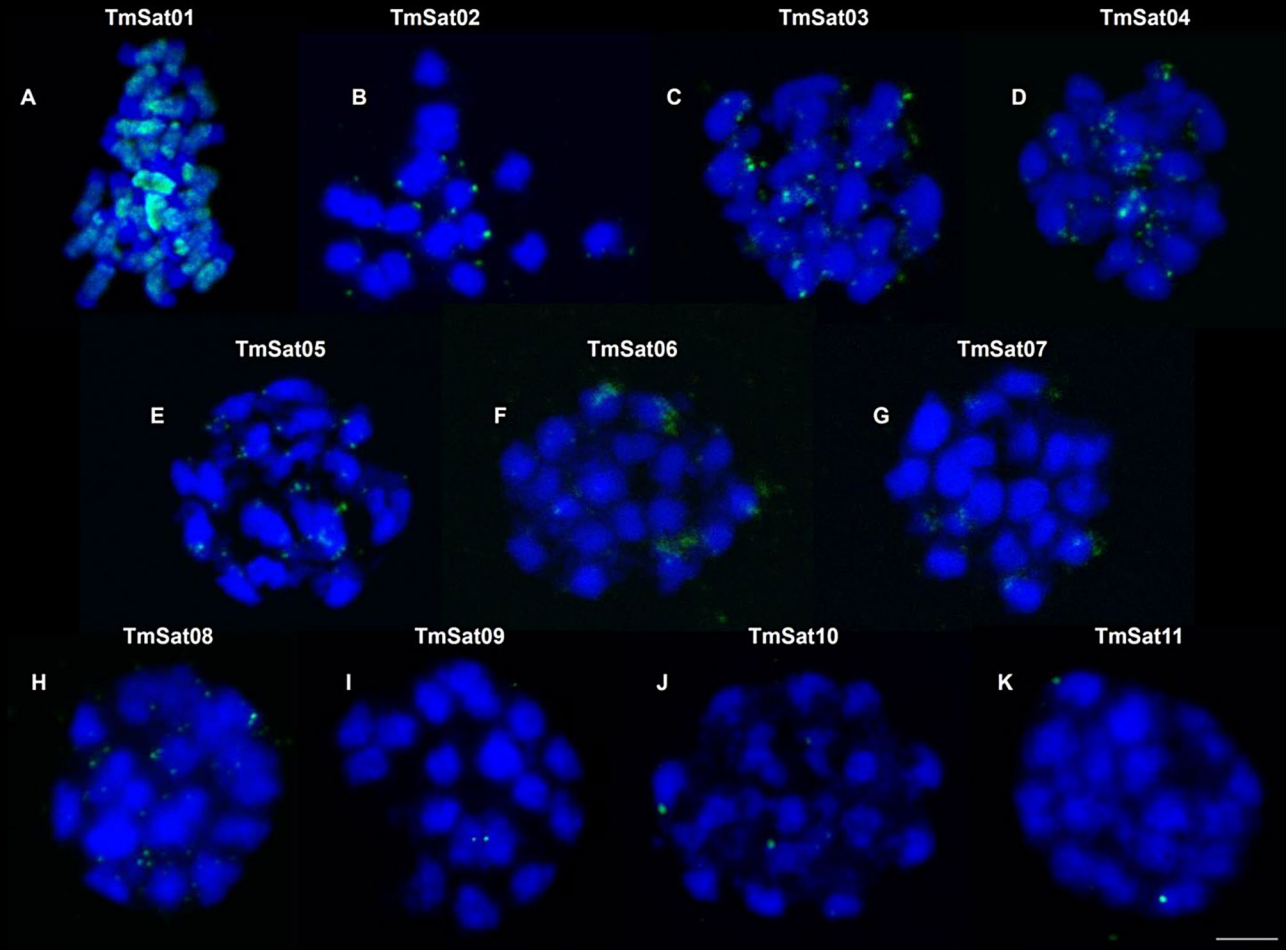
Fluorescence in situ hybridization localization of TmSat01–TmSat11 on *Tenebrio molitor* chromosomes. Localization of satDNA repeats belonging to (**A**) TmSat01, (**B**) TmSat02, (**C**) TmSat03, (**D**) TmSat04, (**E**) TmSat05, (**F**) TmSat06, (**G**) TmSat07, (**H**) TmSat08, (**I**) TmSat09, (**J**) TmSat10, and (**K**) TmSat11. SatDNA signals are shown in green, and chromosomes are counterstained with DAPI (blue). Scale bar represents 3 µm

Fluorescence in situ hybridization (FISH) mapping of satDNAs revealed diverse chromosomal distribution patterns (Fig. 2). TmSat01 encompassed large pericentromeric and interstitial heterochromatic blocks on all chromosomes (Fig. 2A). Probes for TmSat02, 03, 04 and 05 satDNAs produced multiple interspersed signals (Fig. 2B, C, D). TmSat07, 08 also exhibited interspersed pattern of distribution, but with a fewer signals (Fig. 2E, G, H). TmSat06 signals were clustered terminally in two chromosomal pairs in addition to the several weaker and interspersed signals (Fig. 2F). Finally, TmSat09, TmSat10, and TmSat11 each hybridized to a single chromosomal pair, TmSat10 and TmSat11 located terminally (Fig. 2J, K), and TmSat09 positioned centrally on the chromosome (Fig. 2I).

### SatDNA divergence

The evolution of satDNAs is shaped by homogenization processes, which reduce sequence divergence, and the accumulation of mutations, which increase divergence. Consequently, satDNA divergence landscapes provide valuable insights into the structure (and evolution) of sequence variants within a given satDNA family. Peaks at low divergence values are generally interpreted as signatures of recent amplification or active homogenization, whereas peaks at higher divergence values likely represent older or non-homogenized variants that have accumulated mutations over time. We therefore analyzed the divergence profiles of the 11 *T. molitor* satDNAs.

**Fig. 3:**
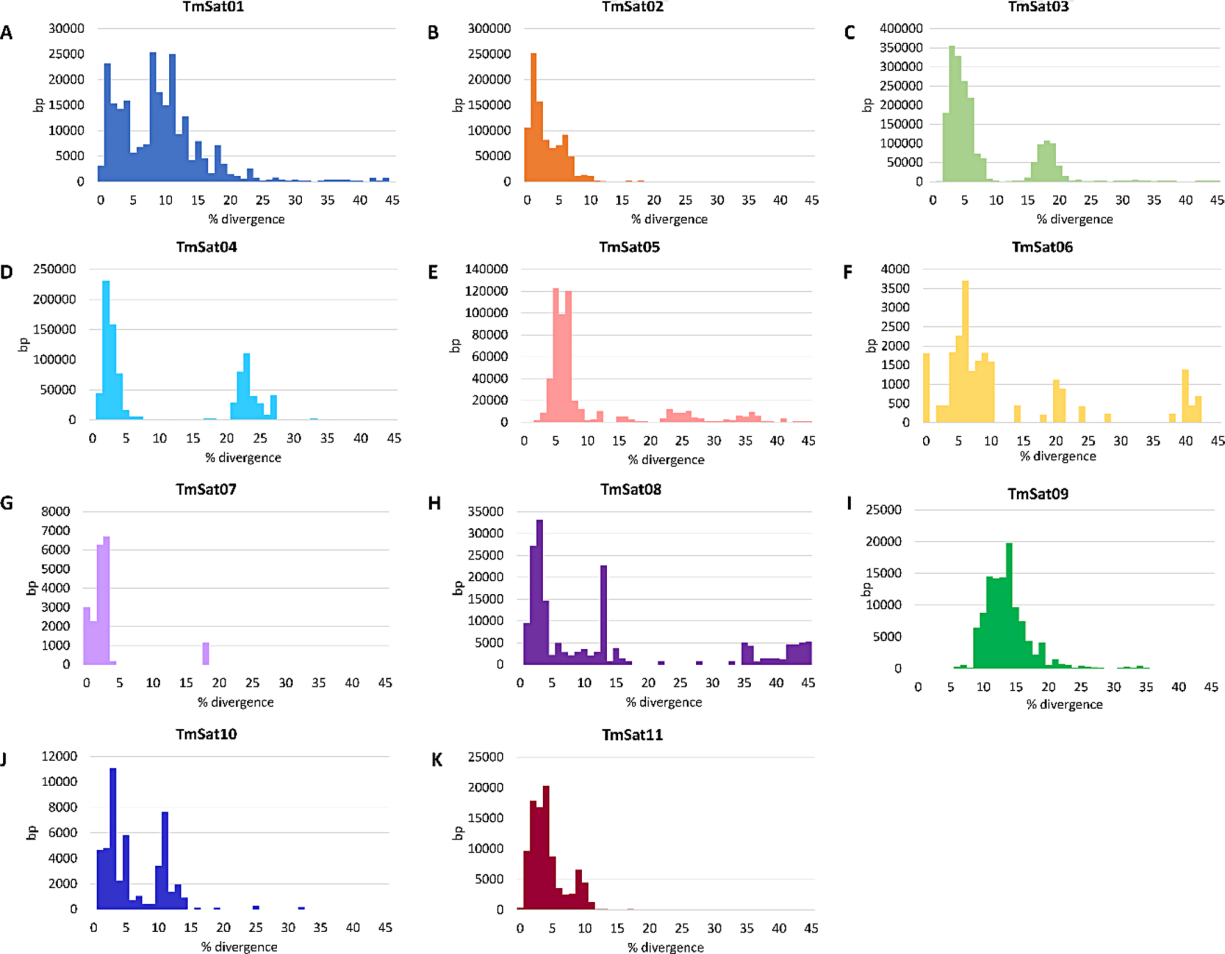
The distribution of sequence divergence for each satDNA calculated relative to its consensus sequence using the Kimura 2-parameter model. The x-axis represents percentage divergence, while the y-axis indicates the satDNA abundance within each divergence class. Panels correspond to: **A**) TmSat01, **B**) TmSat02, **C**) TmSat03, **D**) TmSat04, **E**) TmSat05, **F**) TmSat06, **G**) TmSat07, **H**) TmSat08, **I**) TmSat09, **J**) TmSat10, **K**) TmSat11. SatDNA-specific colors are preserved in Figs. 1, 3 and 7

Figure 3 shows repeat landscape plots illustrating the divergence of individual monomers extracted from chromosome-level genome assemblies from their consensus sequence. TmSat02 and TmSat11 each exhibit a single dominant peak at 0–10% divergence (Fig. 3B, K). For TmSat09 this peak is shifted towards higher divergence values, 10–20% (Fig. 3I). Divergence plots of TmSat03, TmSat04, and TmSat07 display two distinct peaks: a major peak at very low divergence (up to 10%) and a smaller peak at higher divergence values (Fig. 3C, D, G). Several other satDNAs (TmSat01, TmSat05, TmSat06, TmSat07, TmSat08, TmSat10) exhibit a broad range of sequence divergence, with a dominant peak at low divergence accompanied by multiple smaller peaks at higher divergence levels (Fig. 3A, E, F, H, J).

### SatDNAs in extrachromosomal circular DNA (eccDNA)

**Fig. 4:**
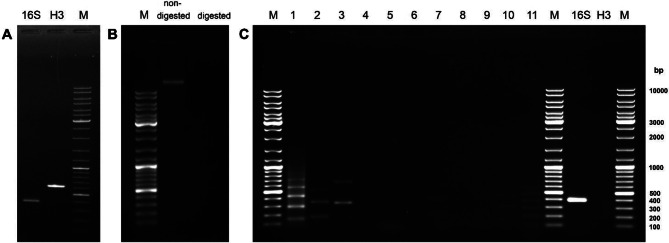
EccDNA analysis. **A**) agarose gel separation of PCR products for 16S and H3 amplification from complete genomic DNA. **B**) equal amounts of non-digested and Exonuclease V-digested genomic DNA loaded on the gel. **C**) PCR amplification of satDNAs from the eccDNA fraction. Numbers 1–11 correspond to TmSat01 – TmSat11. The 16S lane displays amplification, while in H3 lane amplification was absent. M indicates the molecular size marker

One of the mechanisms proposed to be included in the spread of satDNA sequences is via extrachromosomal circular DNA (eccDNA). To investigate this, we isolated and screened eccDNA from *T. molitor* for the presence of the 11 satDNAs. Following total DNA isolation, linear genomic DNA was removed using Exonuclease V (Fig. 4). The absence of linear genomic DNA and the preservation of circular DNA were confirmed using appropriate controls: amplification of H3 (negative control, confirming the absence of linear DNA) and 16S (positive control for circular DNA) is shown in Fig. 4A. The exonuclease digestion result is shown in Fig. 4B. Figure 4C depicts the amplification results from eccDNA fraction. There, 16S amplification was observed, whereas H3 amplification was absent, confirming successful isolation of circular DNA and removal of linear DNA (Fig. 4C). Using this approach, we detected six satDNAs in the eccDNA fraction: TmSat01, TmSat02, TmSat03, TmSat07, TmSat10, and TmSat11, (Fig. 4C).

### SatDNA transcription

**Fig. 5:**
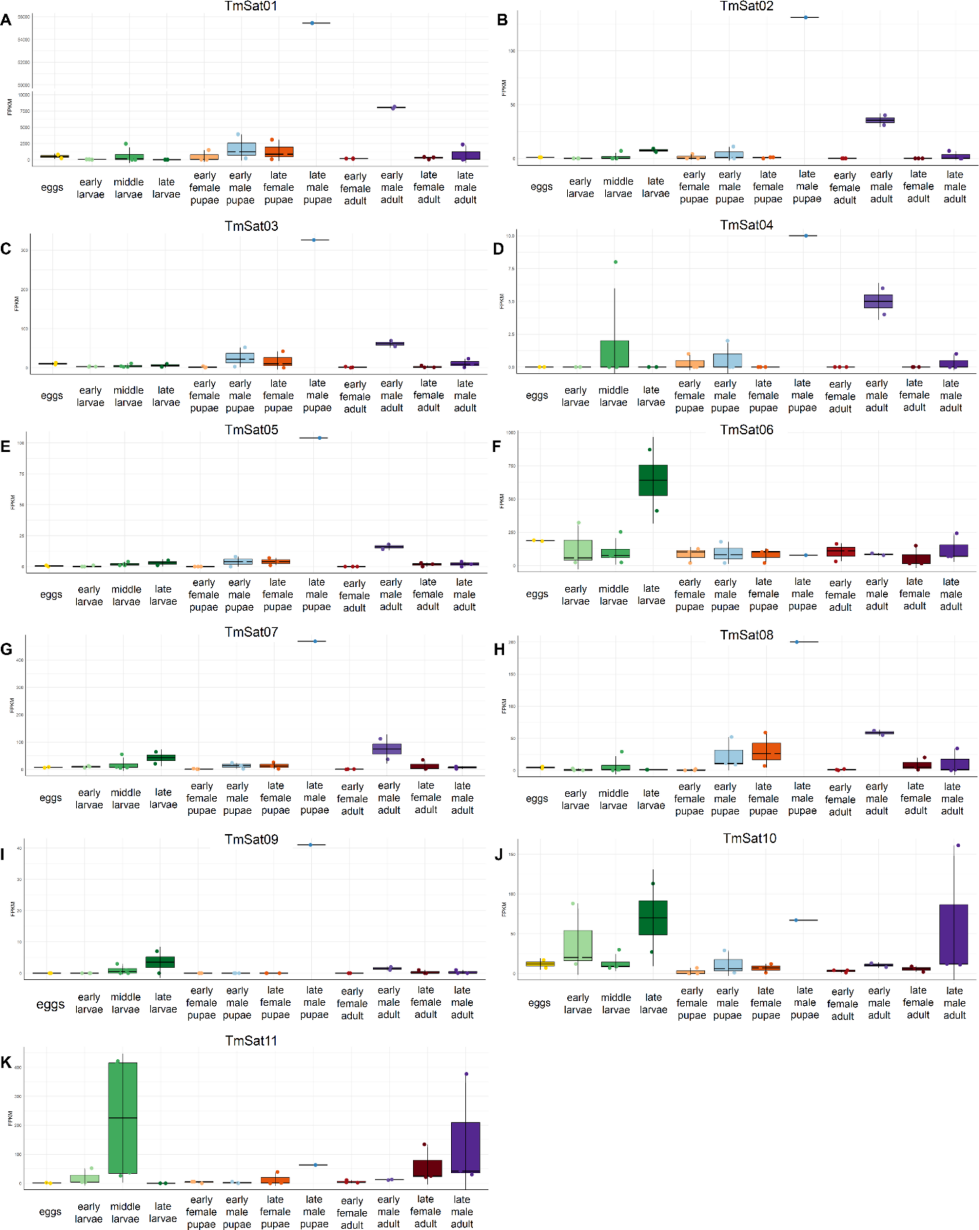
Transcription dynamics of 11 satDNAs of *T. molitor* across different developmental stages in females and males. Each sample is based on biological replicates shown by dots and error bars indicate the standard deviations. Stage-specific color facilitate comparison across developmental stages. Panels correspond to: **A**) TmSat01, **B**) TmSat02, **C**) TmSat03, **D**) TmSat04, **E**) TmSat05, **F**) TmSat06, **G**) TmSat07, **H**) TmSat08, **I**) TmSat09, **J**) TmSat10, **K**) TmSat11

The transcription of all 11 satDNAs was analyzed using RNA-seq libraries from *T. molitor* eggs, early, mid, and late larvae, early and late pupae, and early and late adults of both sexes. Our analyses revealed that all 11 satDNAs are transcribed. For the majority, transcription peaked in late male pupae and was present at reduced levels in early male adults (Fig. 5). TmSat06 showed increased transcription in eggs and late larvae (Fig. 5F), while TmSat10 was most active during both early and late larval stages, as well as in late male pupae and late male adults (Fig. 5J). In contrast, TmSat11 was most strongly transcribed during mid-larval stages and late male pupae, as well as in both late female and male adults (Fig. 5K).

### SatDNA taxonomic distribution, phylogeny and age

**Fig. 6:**
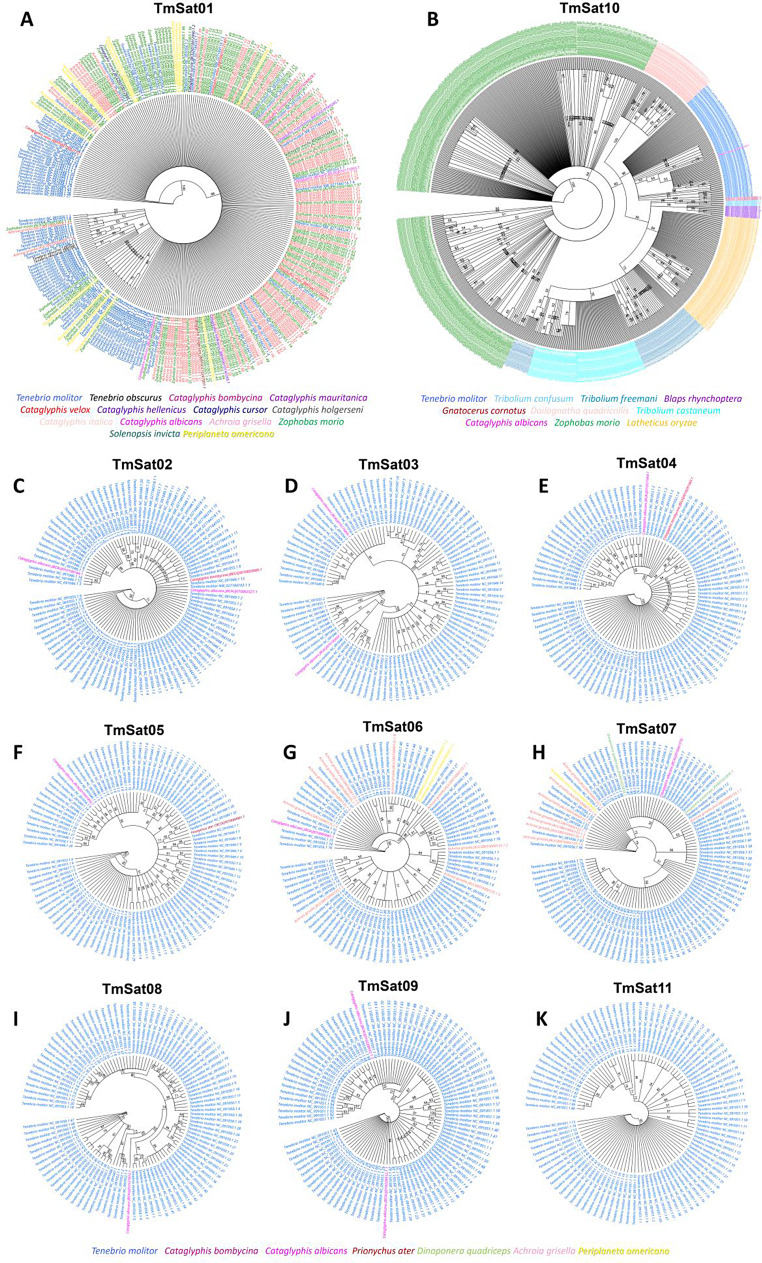
Phylogenetic reconstruction for 11 satDNAs, based on monomer sequences from insect species. Monomer sequences are color-coded according to their species of origin, denoted in the legend. Panels correspond to: **A**) TmSat01, **B**) TmSat10, **C**) TmSat02, **D**) TmSat03, **E**) TmSat04, **F**) TmSat05, **G**) TmSat06, **H**) TmSat07, **I**) TmSat08, **J**) TmSat09, **K**) TmSat11

GenBank databases were scanned using the consensus sequences of 11 *T. molitor* satDNAs to gain insight into their taxonomic distribution. In the Supplementary Table 2, insect orders with available genomic data are presented, while for Coleoptera, the corresponding families are also shown. The BLAST search results revealed hits in 47 species across six insect orders: Coleoptera, Lepidoptera, Hymenoptera, Diptera, Hemiptera, and Blattodea (Supplementary Table S3). Monomers for phylogeny reconstruction were selected and subsampled as described above. The largest number of species and sets of monomers were found with TmSat01 and TmSat10, comprising 320 monomers from 13 species (TmSat01) and 885 monomers from 12 species (TmSat10). TmSat01 sequences did not form distinct clusters and showed no species-specific grouping (Fig. 6A). In contrast, TmSat10 exhibited pronounced clustering, with monomers of each species forming species-specific groups and/or subgroups (Fig. 6B). An exception was a single monomer from *Cataglyphis albicans*, which clustered with those from *T. molitor*. *Tribolium castaneum* and *Tribolium freemani* monomers cluster together, followed by further separation of *T. castaneum* monomers into species-specific group (Fig. 6B). Phylogenetic trees for TmSat02, 03, 04, 05, 08, and 09 were dominated by *T. molitor* monomers, with only limited contributions from *C. albicans*, *C. bombycina*, or *Prionychus ater* (Fig. 6C–F, I, J). In the case of TmSat03 and 09, *T. molitor* monomers form several subgroups. However, monomers from *C. albicans* did not cluster together; instead, they were dispersed among *T. molitor* sequences (Fig. 6D, J). *T. molitor* monomers from TmSat02, 04, 05, and 09 did not form distinct phylogenetic clusters but were observed in numerous clusters and subclusters (Fig. 6C, E, F, J). The phylogenetic tree of TmSat06 included monomers from *Achroia grisella*, *Periplaneta americana*, and *C. albicans* in addition to *T. molitor*. These monomers did not form species-specific groups and were instead scattered throughout the tree, except for those of *P. americana*, which clustered together (Fig. 6G). TmSat07 incorporated an additional species, *Dinoponera quadriceps*, yet similarly lacked species-specific grouping (Fig. 6H). TmSat11 was detected exclusively in *T. molitor* (Supplementary Table S3; Fig. 6K). Approximately half of the monomers selected for phylogenetic analysis present no evident grouping, while the rest form a number of small clusters (Fig. 6K).

**Fig. 7:**
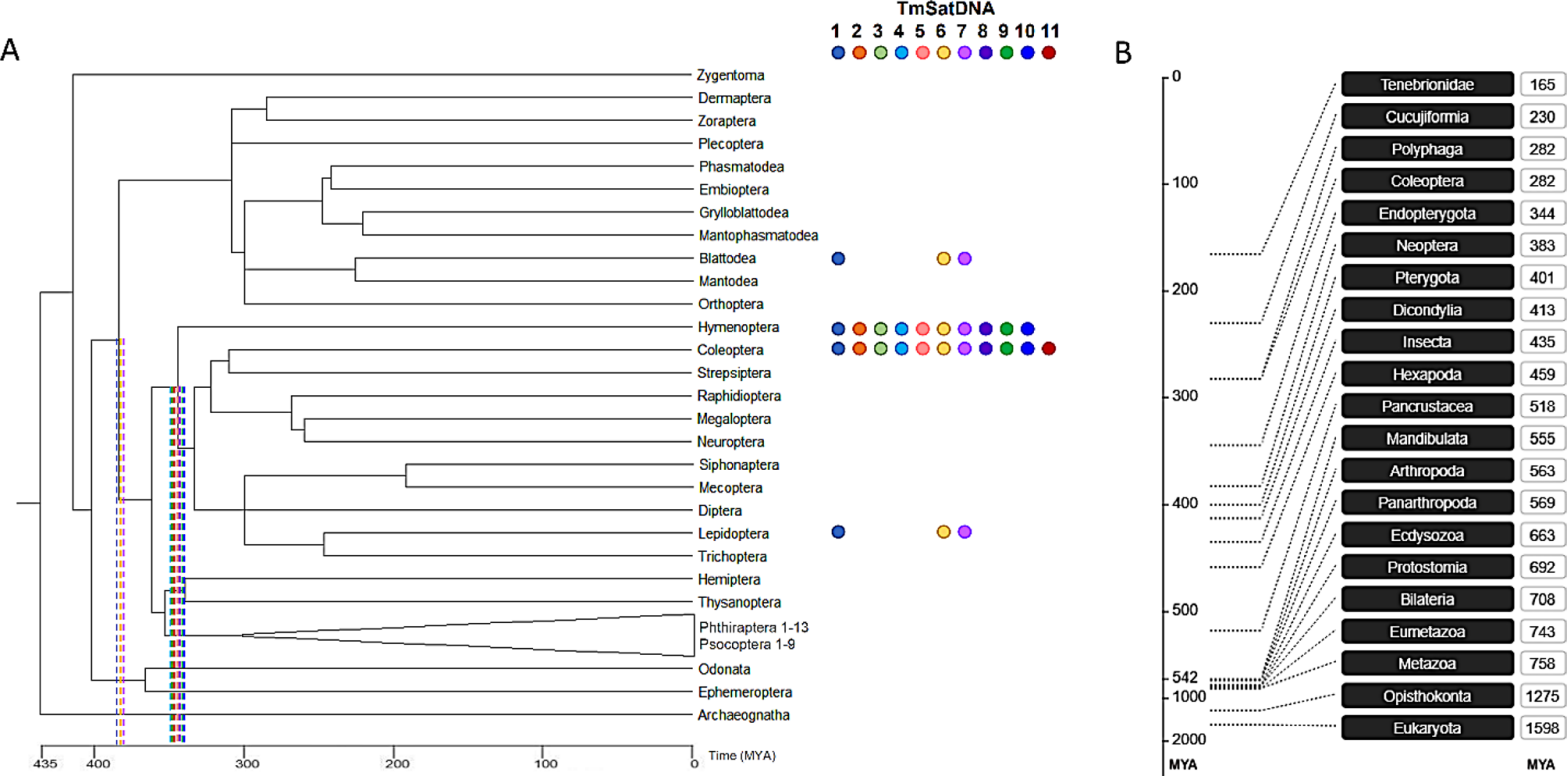
Taxonomic distribution and age estimation of 11 satDNAs (TmSat01–11). **A**) time-calibrated phylogeny of insect orders with annotated occurrence of *T. molitor* satDNAs. Each satDNA is indicated by a distinct color. Circles denote its presence within a given insect order, while dashed lines represent its estimated minimal evolutionary age. **B**) an evolutionary timeline of family Tenebrionidae

We estimated the minimal age of the 11 satDNAs after juxtaposing their distribution against the timetree of insect orders (Fig. 7A). SatDNAs TmSat01, 06, 07 were detected in species belonging to Coleoptera, Lepidoptera, Hymenoptera, and Blattodea. The separation time of this orders dates to ~ 380 MYA, which would denote the minimal age of these satDNAs. The presence of TmSat04 within Coleoptera, Hymenoptera, Diptera, Hemiptera species suggest a minimal age of ~ 360 MYA. The divergence of Hymenoptera from Coleoptera, Lepidoptera and Diptera ~340 MYA marks this timepoint as the minimal age of TmSat02, 03, 05, 08, 09, and 10. Finally, TmSat11, which was detected only in *T. molitor* (Coleoptera), represents the youngest satDNA in our dataset, originating sometime after the emergence of the Tenebrionidae family, which is dated to ~ 165 MYA (Fig. 7B).

## Discussion

Sequence analysis, chromosomal mapping, divergence profiles, phylogenetic analyses and age estimation enabled us to delineate the evolutionary origin and diversification of TmSatDNAs while transcription analysis and eccDNA provided insights into functional and distribution potential of these sequences.

### Diverse distribution patterns of satDNA sequences

A massive difference in satDNA monomer counts between the two assembly versions has been observed, particularly for TmSat01. The difficulties arise in reconstructing exact sequential order and length of segments composed of highly similar repeat units present in high copy-numbers. Consequently, repetitive sequences are frequently omitted from datasets available in public databases, or even significant errors in genome annotation are introduced [35, 52]. Highly repetitive sequences often suffer from repeat collapse during genome assembly, where multiple copies are erroneously merged into one or certain regions are left as gaps in the final assembly [53]. This indicates that even chromosome-level assemblies may be incomplete and/or misassembled, a problem that is particularly pronounced in species with large genomic blocks composed of highly abundant tandemly repeated satDNA sequences, such as *T. molitor* TmSat01. For example, genome assembly of *T. molitor* is only ~400 Mb while cytological analysis estimated the actual genome size to ~ 780 Mb [34]. Therefore, relying solely on a (single) assembly can lead to a severe underestimation in the amount of major satDNA, or its positioning, masking their influence on the genome architecture and/or potential functions. The satDNA distribution patterns inspected in situ and in silico are largely consistent for most satellites, with the most notable discrepancies observed for TmSat01, TmSat06, and TmSat07. Although TmSat01 is known to represent the major satDNA of *T. molitor*, forming large pericentromeric blocks [54, 55], Fig. 2A), its in silico representation is significantly reduced, appearing only as numerous short arrays frequently interrupted by assembly gaps. On the other hand, TmSat06 and TmSat07 are detected exclusively on the Y chromosome in the genome assembly, whereas FISH reveals their presence on additional chromosomes (Fig. 2). It is important to highlight that the satDNA research benefits from complementing and juxtaposing in situ and in silico localizations, as their capacity in displaying the localization of long arrays and short satDNA segments are fundamentally opposite [4]. While very short arrays and interspersed monomers are likely to fall below the detection threshold of FISH, they will mostly be successfully included in the genome assembly. Oppositely, long arrays are mostly excluded or reduced in the assembly, while in the FISH analysis they display strong clustered signals [4]. While TmSat01 forms large heterochromatic blocks and is present on all chromosomes of *T. molitor*, TmSat09, 10, and 11 are restricted to only a single chromosome pair (Fig. 2, Table 1). Similar examples of chromosome-specific satDNAs have been reported in other taxa, such as the DBC-150 satellite family in *Drosophila buzzatii* [56], the MCSAT family in *Muscari* species [57], and OocSat42 in the flea beetle *Omophoita octoguttata* [58], and others. Such confinement of a satDNA to a single chromosome may result from several factors, including the relatively recent origin of the satDNA, or structural or functional constraints. In addition, reduced recombination, limited amplification, or low rates of transposition-mediated dispersal may further contribute to a limited spread of a satDNA across the genome. *T. molitor* telomeres are known to be composed of TCAGG telomeric repeats [59], which are omitted from the nucleotide sequence of TmSat02 and TmSat04. Nevertheless, based on their predominantly terminal localization on chromosomes in situ and in silico, TmSat02 and TmSat04 contribute to the (sub)telomeric regions structurally, and potentially even functionally.

In comparison to the consensus-based annotation, *de novo* detection using TideHunter recovered nine of the eleven known satDNAs (Supplementary Figure S4). This observation reinforces the well-established advantages of assembly-free approaches, particularly when complemented by subsequent annotation in genome assemblies. Conversely, the utility of the assembly-based approach is illustrated by the detection of several repeats in additional TRCs that are predominantly present in the genome as a single array (Supplementary Figure S4B).

### Distinct turnover dynamics and homogenization efficiencies for each *T. molitor *satDNA

The evolution of satDNAs is shaped by homogenization processes, such as unequal crossing-over, gene conversion, and eccDNA-mediated turnover, which act to maintain sequence similarity among monomers within a family [6, 16, 60]. In *T. molitor*, low divergence among certain satDNA families and different patterns in divergence landscapes presented in Figure 3 indicate the action of these processes in shaping their genomic organization and evolutionary dynamics. The divergence profiles of the 11 satDNAs differ from one another, (Fig. 3), indicating the lack of strong homogenization processes that would affect all satDNAs equally. Sequences with low divergence values exhibit minimal deviation from the consensus sequence, reflecting either well preserved copies, or recently amplified/homogenized copies (TmSat02 and TmSat11). High sequence preservation could also be a sign of functional significance of a particular sequence. In contrast, the sequences positioned at higher divergence values represent more degenerate copies with accumulated mutations (TmSat09). Multiple peaks (observed for TmSat01, TmSat05, TmSat06, TmSat07, TmSat08, TmSat10) could be a result of a more intensive propagation of several sequence variants forming subgroups that are homogenous within. Although homogenization is generally expected to be more effective locally, within a given heterochromatic block, array, or chromosome, this is not necessarily the rule. For example, TmSat02 is distributed across a large number of chromosomes (Table 1, Fig. 2) yet retains high sequence identity (Fig. 3). TmSat09, TmSat10, and TmSat11 are each confined to a single chromosome pair (Table 1, Fig. 2), while exhibiting different divergence patterns (Fig. 3I, J, K). Together, these findings suggest distinct turnover dynamics and homogenization efficiencies for each satDNA in *T. molitor*.

### The presence of satDNA in the eccDNA

EccDNA plays a significant role in the dynamics, propagation, and homogenization of satDNA. EccDNAs can originate from chromosomal DNA through several mechanisms, including recombination, DNA repair, and aberrant replication. In the case of satDNA, eccDNAs are frequently produced by intra-strand recombination within tandem arrays [16, 61]. Once formed, eccDNAs may serve as templates for rolling-circle replication, resulting in the amplification of satDNA repeats. This process can facilitate the homogenization of satDNA by enabling the redistribution of identical or nearly identical monomers across the genome.

Traditional detection of satDNAs in the eccDNA fraction relied on using two-dimensional agarose gel electrophoresis followed by Southern hybridization with probes for a specific satDNA (e.g. [15, 61, 62]). Through different and novel approach, based on eccDNA isolation and PCR amplification, we have confirmed the existence of six satDNAs in the eccDNA fraction, TmSat01, 02, 03, 07, 10 and 11 (Fig. 4). While the functional role of eccDNA in satDNA in turnover requires further structural and quantitative verification, we hypothesize some potential scenarios. As each of these satDNAs presents distinct abundance, distribution and chromosomal occupancy, their presence in the eccDNA could be related to different occurrences. For some, eccDNA could be a mean of propagation and distribution across the genome/chromosomes. For the others, it could be merely an instrument for array shortening via excision, or of the array prolonging via circle formation, amplification, and reintegration. TmSat10 and 11 are present in the eccDNA fraction, but still remain limited to one chromosome each, chromosome 1 (TmSat10) and 6 (TmSat11) (Table 1, Fig. 2). This pattern could also indicate that satDNA propagation via eccDNA is not a random process. Successful reintegration of eccDNA-derived satDNA potentially requires a certain “seed” of the corresponding satDNA to be present. Transposition-mediated spread of satDNAs could serve as satDNA seed distribution, enabling subsequent eccDNA-driven expansion. Alternatively, certain satDNAs may be constrained by functional or structural requirements that restrict their presence to specific chromosomes or genomic regions. In these cases, eccDNA may serve primarily to modulate array length rather than to promote genome-wide dispersal.

### Stage- and sex-dependent transcription of *T. molitor* satDNAs

There is increasing evidence that satDNAs undergo active and tightly regulated transcription, and that their transcripts play important roles in a variety of cellular processes, including heterochromatin formation, centromere function, and the regulation of gene expression across various biological contexts (reviewed in [11]). Although transcription of satDNAs in insects has been investigated within various orders [12, 59, 63–69], etc.), comprehensive studies covering all satDNA of the species’ satellitome, spanning at the same time different tissues and/or developmental stages, are still scarce.

Increased transcription for majority of satDNAs in late male pupae and early male adults of *T. molitor* indicates the role of satDNAs in development, with special focus on the male lineage (Fig. 5). For several satDNAs, transcription is also observed at the larval stage, which may likewise reflect contributions from the male lineage, as the sex of those developmental stages could not be distinguished. The majority of satDNAs present similar expression profiles, indicating their simultaneous activation, with a clear association to specific developmental timepoints. De Lima et al. [12] analyzed transcription of satDNAs pBuM (in *Drosophila mojavensis*) and CDSTR198 (in *D. buzzatii*) across embryos, larvae, pupae, and adult males and females, observing the highest levels in pupae and adult males. In *Rhodnius prolixus*, Montiel et al. [13] found that 33/39 satDNAs are transcribed and can be categorized into four expression patterns (ubiquitous, antenna-enriched, gonad-enriched, and gonad-specific), with some satDNAs showing pronounced sex/tissue biases. Our results, as well as other studies indicate that satDNA transcription in insects seems to be widespread and regulated, but varies depending on the satDNA family, tissue, developmental stage, or biological context.

### Contrasting phylogenetic distributions of *T. molitor* satDNAs

The clustering patterns of satDNA sequences in the phylogenetic analyses reflect their evolutionary trajectories within and between species. TmSat01 and TmSat10 were detected in a larger number of species compared to other satDNAs, yet they exhibit contrasting phylogenetic clustering patterns. While TmSat01 sequences do not form species-specific groups, TmSat10 shows more pronounced species-specific clustering (Fig. 6). The monomer clustering observed in the TmSat10 phylogeny suggests that these sequences have evolved independently within specific lineages, accumulating mutations over time and diverging from the ancestral sequence. Similarly, the BIV160 satDNA family, present in various bivalve species, shows a shared origin that subsequently diverged to produce species-specific variants [70]. In contrast, TmSat01 and several other satDNAs lack clear species-specific clustering, which could indicate preservation of the ancestral sequence. Comparable patterns of long-term sequence conservation have been observed in alligators and caimans, where a small number of satDNA families exhibit little variation both within and between species [71]. Alternatively, random distribution of mutations preventing clear separation, or even horizontal transfer events could lead to a mixed satDNA monomer distribution in the phylogenetic tree.

### Age of satDNA sequences

For some of the *T. molitor* satDNAs, age estimation was previously performed in Oppert et al. [34]. However, the continuous expansion of NCBI GenBank with newly sequenced insect genomes allowed us to obtain an updated and more comprehensive overview of satDNA species-distribution. TmSat01, 06, and 07 represent the oldest satDNAs in the *T. molitor* genome, with a minimal age of ~ 380 MYA. TmSat01 shows broad distribution both chromosomally (forming large pericentromeric blocks on all chromosomes of *T. molitor*) and across species, being present in numerous insect genomes (Supplementary Table S3). In contrast, TmSat06 and TmSat07 are restricted to a limited number of chromosomes and are found in only a few currently available insect genomes. TmSat11 is detected exclusively in *T. molitor*, suggesting that it emerged after the speciation of this species. This estimates its age to less than 165 MYA, corresponding to the proposed age of the Tenebrionoidea [72]. Simultaneously, this enables the potential usage of TmSat11 sequence as an alternative marker for species confirmation, particularly relevant in the context of the integration of *T. molitor* into the animal feed and human food market.

The library hypothesis posits that closely related species share a common set of satDNAs inherited from a common ancestor [73]. However, novel satDNAs can arise independently in each species, while existing ones may undergo degeneration, resulting in partially overlapping yet divergent satDNA content across species. This partially explains the variable presence or absence of *T. molitor* satDNAs in different insect species/orders (Fig. 7). At the same time, despite the growing number of available insect genomes (Supplementary Table S2), *T. molitor* satDNAs were detected in only a limited number of species (Supplementary Table S3). The absence of detection does not imply true absence, but rather reflects current database limitations and/or assembly challenges, particularly for long tandem arrays

It should be noted that satDNA repeats are often integral components of transposable elements [74–78]. In line with this, the conserved regions flanking the TmSat06 and TmSat07 arrays, although currently unclassified, may represent segments of transposable elements. Additionally, eccDNAs have been shown to act as vehicles for the transport of genetic material [79, 80]. In this context, the patchy phylogenetic distribution of these satDNAs, combined with higher sequence similarity than expected from vertical inheritance alone (Supplementary Table S3, Fig. 7), could suggest even the occurrence of horizontal transfer events, potentially mediated by transposable elements and/or eccDNAs.

## Conclusion

This study provides an integrative evolutionary framework for understanding satDNA dynamics in the yellow mealworm *Tenebrio molitor*. By combining cytogenetics, comparative genomics, phylogenetics, divergence profiling, eccDNA screening, and transcriptional analyses, we demonstrate that individual satDNA families follow distinct evolutionary trajectories shaped by differential homogenization, turnover rates, chromosomal context, and lineage history. Our results reveal that satDNAs range from deeply conserved families spanning multiple insect orders to lineage-specific satDNAs restricted to *T. molitor*, reflecting both ancient origins and recent emergence. The detection of multiple satDNAs in eccDNA fractions supports a role for eccDNA in satDNA turnover and genome (re)shaping, while coordinated, stage-specific transcription highlights their potential functional relevance during development. Together, these findings emphasize that satDNAs are dynamic, functionally relevant components of eukaryotic genomes and underscore the importance of such comprehensive, multi-level analyses for elucidating their evolutionary significance.

## Electronic supplementary material

Below is the link to the electronic supplementary material.

Supplementary material 1

## Data Availability

The data underlying this article are available in the article and in its online supplementary material. Consensus sequences of the 11 *T. molitor* satDNAs are deposited in NCBI under accession numbers PX395431–PX395441.
