# Supplementary material for: Integrative satellitomics reveals distinct patterns of organization, transcription and evolution of satellite DNAs in Tenebrio molitor
Source: Biol Direct. 2026 May 8;21:74. doi: 10.1186/s13062-026-00808-1 (PMC13192020; doi:10.1186/s13062-026-00808-1)
Supplement: Supplementary file 1 — Supplementary material 1 [file 13062_2026_808_MOESM1_ESM.docx]

**Supplementary Materials**

**Integrative satellitomics reveals distinct patterns of organization, transcription and evolution of satellite DNAs in *Tenebrio molitor***

Patrik Majcen ^1^, Antonio Sermek ^1^, Đurđica Ugarković ^1^, Brenda Oppert ^2,3^,

Miroslav Plohl ^1^, Eva Šatović-Vukšić ^1^*

^1^ Ruđer Bošković Institute, Bijenička 54, 10000 Zagreb, Croatia

^2^ Kansas State University, Manhattan, KS 66502, USA

^3^ Entovations LLC, Manhattan, KS 66503, USA

*correspondence: [esatovic@irb.hr](mailto:esatovic@irb.hr)


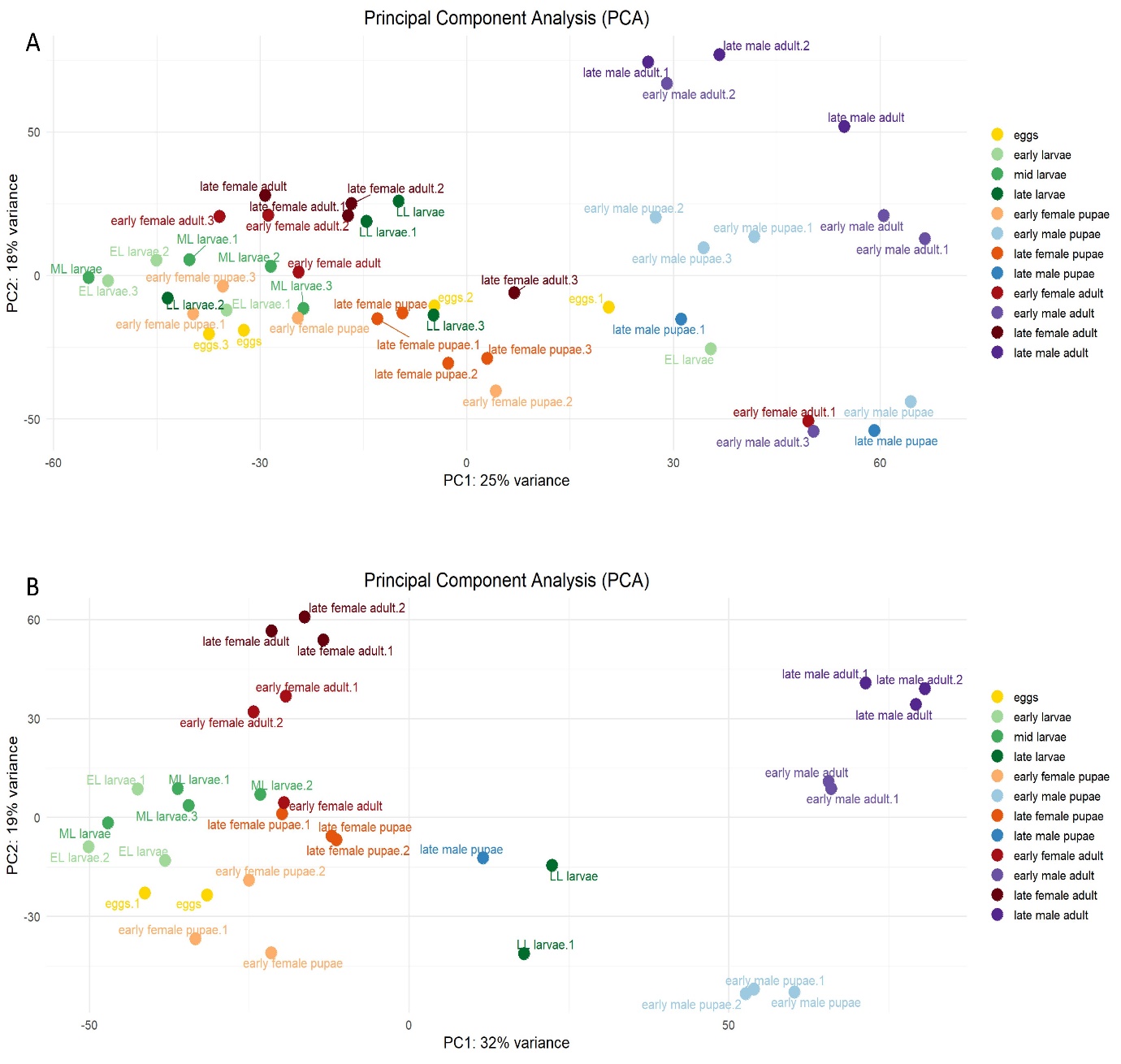


Supplementary Figure S1. Principal component analyses of RNA-Seq datasets belonging to different developmental stages of *T. molitor* before (a) and after (b) outlier removal.


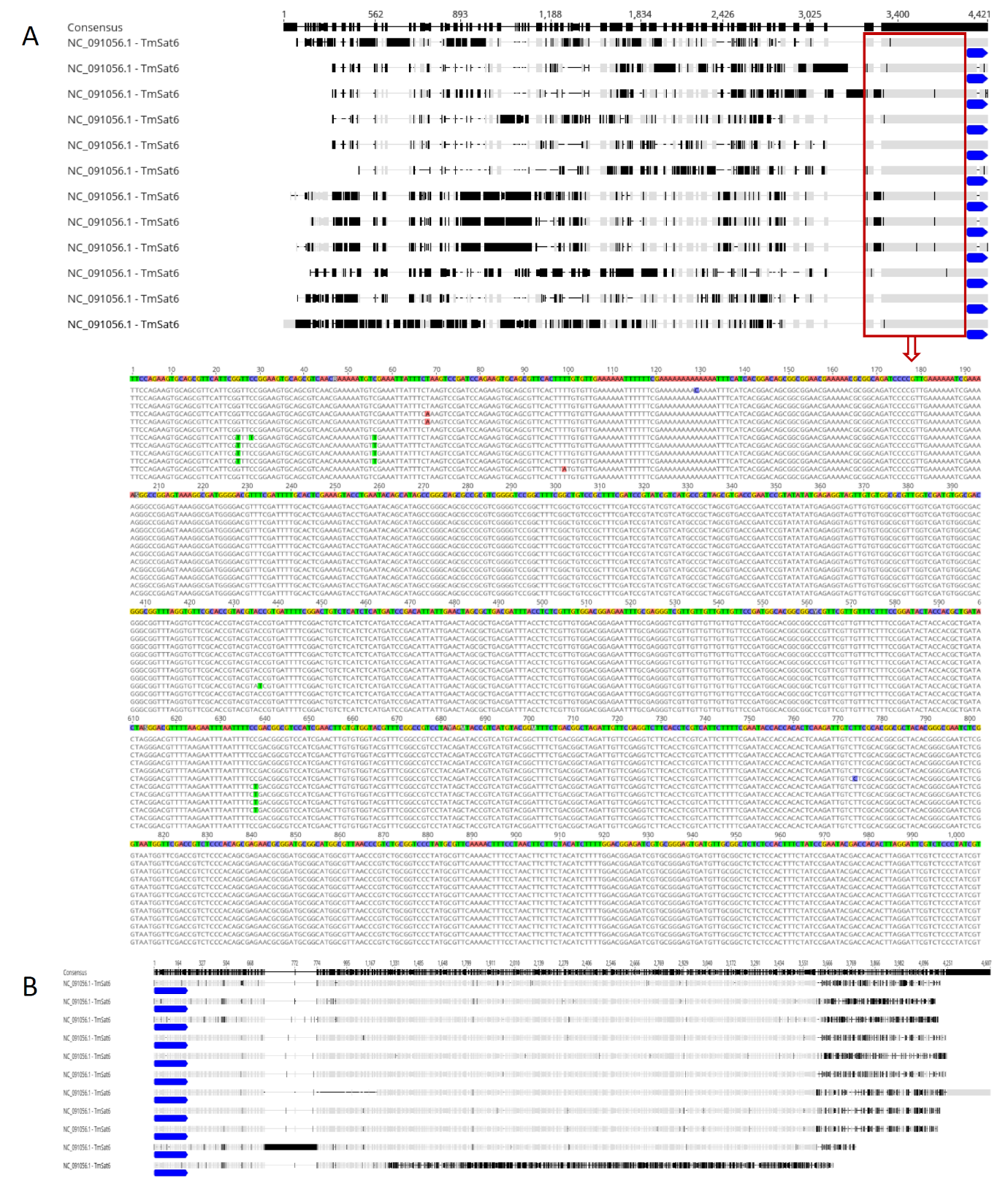


Supplementary Figure S2. Nucleotide alignments of sequence segments preceding (A) and following (B) TmSat06 arrays. Blue arrows in represent the first (A) or the last (B) monomer of TmSat06 arrays. Highly conserved segment of 1,013 bp directly preceding TmSat06 arrays is marked with a red box with the extraction of the nucleotide sequences alignment displayed below.


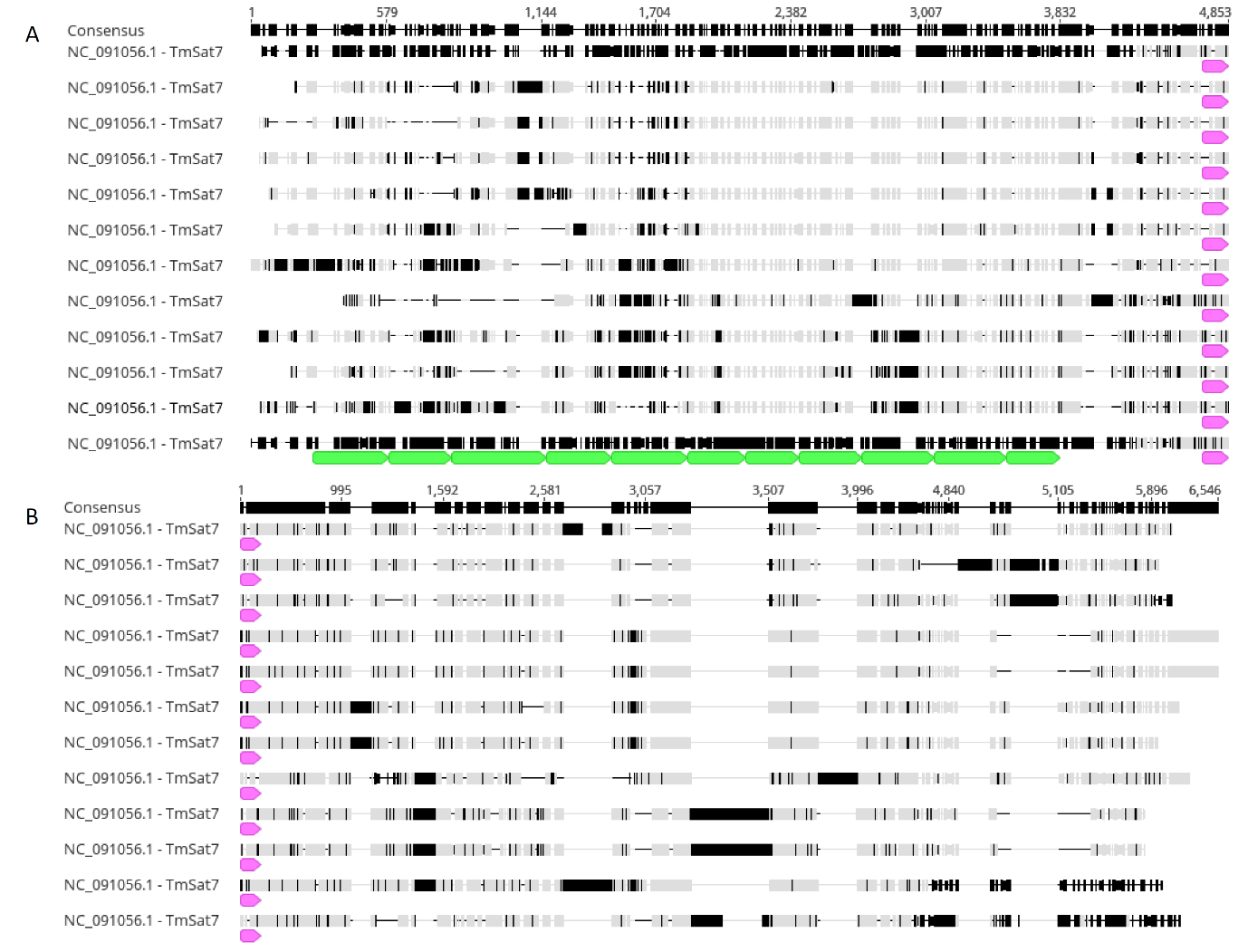


Supplementary Figure S3. Nucleotide alignments of sequence segments preceding (A) and following (B) TmSat07 arrays. Pink arrows in represent the first (A) or the last (B) monomer of TmSat07 arrays. Green arrows represent TmSat05 monomers in one of the sequence segments.

Supplementary Figure S4. The output of *de novo* detection of satDNAs in the *T. molitor* reference genome assembly using TideHunter. Information for tandem repeat clusters (TRCs) identified from the genome assembly is shown. Seven satDNAs (TmSat01, TmSat02, TmSat03, TmSat04, TmSat05, TmSat08, and TmSat11) were recovered within the main TRCs (A). Additional clusters contained sequences corresponding to TmSat08, TmSat09, TmSat10, and additional repeats predominantly present in one array (B).

Supplementary Table S1. Primers and parameters used for PCR labelling of FISH probes.

| Primer | Sequence (5’ – 3’) | Initial denaturation | Amplification (35 cycles) | Final extension |
| --- | --- | --- | --- | --- |
| TmSat01_F  TmSat01_R | GAATTCTGTAGTTCTTGCG  CAGGTTCCAAGACGC | 94°C 5' | 94°C 30''  48 °C 30'' 72°C 20'' | 72°C 5' |
| TmSat02_F  TmSat02_R | CGTAAGTTTCAATTATAACTCG  AGAATTTCCTGCCCAAAC |  |  |  |
| TmSat03_F  TmSat03_R | CCATAGCTCCCTTATTAAG  GCGTTTAAATGATTTTCCG |  |  |  |
| TmSat04_F  TmSat04_R | CTACGGCAATGAATTTTAC  CTTGCTTAATAACGGAGC |  |  |  |
| TmSat05_F TmSat05_R | ATTTCCAATAGAACATACCC  GATGTTACAGACTATGCTG |  |  |  |
| TmSat06_F  TmSat06_R | GATGATGTTGAACTAGACG  AGTAGAGCCAACGACC |  |  |  |
| TmSat07_F  TmSat07_R | CGTGCCCTTACGAAC  AATATTATTTTGGAAACGGC |  |  |  |
| TmSat08_F  TmSat08_R | ATGTTTGTGGGTGAAATC  AAACATCTTCCTATCGAC |  |  |  |
| TmSat09_F  TmSat09_R | CAAGTTGTTGGATGTCAG  TAATTGATCCTGGAACCC |  |  |  |
| TmSat10_F  TmSat10_R | CTTCAGTTTTGAGTTTAATTTC  TTGTCGTAAAAGAGAAAAC |  |  |  |
| TmSat11_F  TmSat11_R | GGAGCTCGAAGTTTCG  ACTGAATCTACCACTCC |  |  |  |

Supplementary Table S2. Overview of insect orders with available genomic data, including families for Coleoptera.

| **Order** | **Family** | **No of seq. genomes** |
| --- | --- | --- |
| Archaeognatha |  | **1** |
| Blattodea (cockroaches & termites) |  | **23** |
| Coleoptera (beetles) |  | **790** |
|  | Acanthocnemidae | 0 |
|  | Aderidae | 0 |
|  | Agapythidae | 0 |
|  | Agyrtidae | 0 |
|  | Alexiidae | 0 |
|  | Amphizoidae (trout-stream beetles) | 0 |
|  | Anamorphidae | 0 |
|  | Anischiidae | 0 |
|  | Anthicidae (ant beetles) | 2 |
|  | Anthribidae (fungus weevils) | 5 |
|  | Apionidae | 6 |
|  | Archeocrypticidae | 0 |
|  | Artematopodidae | 0 |
|  | Aspidytidae | 0 |
|  | Attelabidae (leaf-rolling weevils) | 2 |
|  | Belidae | 0 |
|  | Belohinidae | 0 |
|  | Biphyllidae (false skin beetles) | 2 |
|  | Boganiidae | 0 |
|  | Bolboceratidae | 0 |
|  | Boridae | 0 |
|  | Bostrichidae (horned powder-post beetles) | 1 |
|  | Bothrideridae (dry bark beetles) | 1 |
|  | Brachyceridae | 0 |
|  | Brachypsectridae | 0 |
|  | Brentidae (straight-snouted weevils) | 2 |
|  | Buprestidae (jewel beetles) | 7 |
|  | Byrrhidae (pill beetles) | 0 |
|  | Byturidae (fruitworm beetles) | 0 |
|  | Callirhipidae | 0 |
|  | Cantharidae (soldier beetles) | 30 |
|  | Carabidae (ground beetles) | 60 |
|  | Caridae | 0 |
|  | Cavognathidae | 0 |
|  | Cebrionidae | 0 |
|  | Cephaloidae | 0 |
|  | Cerambycidae (long-horned beetles) | 39 |
|  | Cerasommatidiidae | 0 |
|  | Cerophytidae | 0 |
|  | Cerylonidae (minute bark beetles) | 0 |
|  | Chaetosomatidae | 0 |
|  | Chalcodryidae | 0 |
|  | Chelonariidae | 0 |
|  | Chrysomelidae (leaf beetles) | 124 |
|  | Ciidae | 0 |
|  | Clambidae | 0 |
|  | Cleridae (checkered beetles) | 4 |
|  | Cneoglossidae | 0 |
|  | Coccinellidae (ladybird beetles) | 46 |
|  | Corylophidae (minute fungus beetles) | 0 |
|  | Cryptophagidae (silken fungus beetles) | 2 |
|  | Cucujidae (flat bark beetles) | 2 |
|  | Cupedidae (reticulated beetles) | 1 |
|  | Curculionidae (weevils) | 95 |
|  | Cyclaxyridae | 0 |
|  | Dascillidae | 2 |
|  | Decliniidae | 0 |
|  | Dermestidae (skin and larder beetles) | 2 |
|  | Derodontidae | 0 |
|  | Diphyllostomatidae | 0 |
|  | Discolomatidae | 0 |
|  | Dryopidae | 0 |
|  | Dytiscidae (predacious diving beetles) | 2 |
|  | Elateridae (click beetles) | 38 |
|  | Elmidae (riffle beetles) | 2 |
|  | Endecatomidae | 0 |
|  | Endomychidae (handsome fungus beetles) | 2 |
|  | Epimetopidae | 0 |
|  | Erirhinidae | 2 |
|  | Erotylidae (pleasing fungus beetles) | 0 |
|  | Eucinetidae | 0 |
|  | Eucnemidae | 2 |
|  | Eulichadidae | 0 |
|  | Georissidae | 0 |
|  | Geotrupidae (earth-boring dung beetles) | 5 |
|  | Glaphyridae | 0 |
|  | Glaresidae | 0 |
|  | Gyrinidae (whirligig beetles) | 0 |
|  | Haliplidae (crawling water beetles) | 0 |
|  | Helophoridae | 0 |
|  | Helotidae | 0 |
|  | Heteroceridae | 0 |
|  | Histeridae (clown beetles) | 2 |
|  | Hobartiidae | 0 |
|  | Hybosoridae | 0 |
|  | Hydraenidae | 0 |
|  | Hydrochidae | 1 |
|  | Hydrophilidae (water scavenger beetles) | 8 |
|  | Hydroscaphidae | 0 |
|  | Hygrobiidae (diving beetles) | 0 |
|  | Iberobaeniidae | 0 |
|  | Ithyceridae | 0 |
|  | Jacobsoniidae | 0 |
|  | Jurasaidae | 0 |
|  | Kateretidae (short-wingedflower beetles) | 2 |
|  | Laemophloeidae (lined flat bark beetles) | 0 |
|  | Lampyridae (fireflies) | 8 |
|  | Latridiidae (minute brown scavenger beetles) | 2 |
|  | Leiodidae (round fungus beetles) | 5 |
|  | Lepiceridae | 0 |
|  | Limnichidae | 0 |
|  | Lophocateridae | 0 |
|  | Lucanidae (stag beetles) | 7 |
|  | Lutrochidae | 0 |
|  | Lycidae (net-winged beetles) | 1 |
|  | Lymexylidae (ship timber beetles) | 0 |
|  | Mauroniscidae | 0 |
|  | Melandryidae | 2 |
|  | Meloidae (blister beetles) | 12 |
|  | Melyridae (soft-winged flower beetles) | 4 |
|  | Meruidae | 0 |
|  | Metaxinidae | 0 |
|  | Micromalthidae | 0 |
|  | Monommidae | 0 |
|  | Monotomidae (root-eating beetles) | 0 |
|  | Mordellidae (tumbling flower beetles) | 0 |
|  | Mycetophagidae | 4 |
|  | Mycteridae | 0 |
|  | Myraboliidae | 0 |
|  | Nemonychidae | 0 |
|  | Nitidulidae (sap-feeding beetles) | 5 |
|  | Nosodendridae | 0 |
|  | Noteridae (burrowing water beetles) | 0 |
|  | Ochodaeidae | 0 |
|  | Oedemeridae | 2 |
|  | Omalisidae | 0 |
|  | Omethidae | 0 |
|  | Ommatidae | 0 |
|  | Orsodacnidae | 0 |
|  | Passalidae | 0 |
|  | Passandridae (parasitic flat bark beetles) | 0 |
|  | Peltidae | 0 |
|  | Perimylopidae | 0 |
|  | Phalacridae (shining flower beetles) | 0 |
|  | Phengodidae (glowworms) | 1 |
|  | Phloeostichidae | 0 |
|  | Phloiophilidae | 0 |
|  | Phycosecidae | 0 |
|  | Plastoceridae | 0 |
|  | Pleocomidae | 0 |
|  | Podabrocephalidae | 0 |
|  | Priasilphidae | 0 |
|  | Prionoceridae | 0 |
|  | Promecheilidae | 0 |
|  | Propalticidae | 0 |
|  | Prostomidae (jugular-horned beetles) | 0 |
|  | Protelmidae | 0 |
|  | Protocucujidae | 0 |
|  | Protopeltidae | 0 |
|  | Psephenidae | 0 |
|  | Pterogeniidae | 0 |
|  | Ptiliidae (featherwing beetles) | 0 |
|  | Ptilodactylidae | 0 |
|  | Ptinidae | 4 |
|  | Pyrochroidae | 4 |
|  | Pythidae | 0 |
|  | Rentoniidae | 0 |
|  | Rhadalidae | 0 |
|  | Rhagophthalmidae | 1 |
|  | Rhinorhipidae | 0 |
|  | Rhipiceridae | 0 |
|  | Ripiphoridae | 2 |
|  | Salpingidae | 2 |
|  | Scarabaeidae (scarab beetles) | 71 |
|  | Schizopodidae | 0 |
|  | Scirtidae (marsh beetles) | 3 |
|  | Scraptiidae | 6 |
|  | Silphidae (carrion beetles) | 8 |
|  | Silvanidae (silvanid flat bark beetles) | 6 |
|  | Smicripidae (palmetto beetles) | 0 |
|  | Spercheidae | 0 |
|  | Sphaeritidae (false clown beetles) | 0 |
|  | Sphaeriusidae (minute bog beetles) | 0 |
|  | Sphindidae (dry-fungus beetles) | 0 |
|  | Staphylinidae (rove beetles) | 74 |
|  | Stenotrachelidae | 0 |
|  | Synchroidae | 0 |
|  | Synteliidae | 0 |
|  | Telegeusidae | 0 |
|  | Tenebrionidae (darkling ground beetles) | 45 |
|  | Tetratomidae | 2 |
|  | Thanerocleridae | 0 |
|  | Throscidae | 0 |
|  | Thymalidae | 0 |
|  | Torridincolidae | 0 |
|  | Trachelostenidae | 0 |
|  | Trachypachidae | 0 |
|  | Trictenotomidae | 0 |
|  | Trogidae | 0 |
|  | Trogossitidae | 0 |
|  | Ulodidae | 0 |
|  | Vesperidae | 0 |
|  | Zeugophoridae | 0 |
|  | Zopheridae (ironclad beetles) | 8 |
| Dermaptera (earwigs) |  | **8** |
| Diptera (flies) |  | **1.666** |
| Embioptera (web-spinners) |  | **1** |
| Ephemeroptera (mayflies) |  | **14** |
| Grylloblattodea (rock crawlers) |  | **0** |
| Hemiptera (true bugs) |  | **325** |
| Hymenoptera (hymenopterans) |  | **1.099** |
| Lepidoptera (moths & butterflies) |  | **3.468** |
| Mantodea (mantids) |  | **8** |
| Mantophasmatodea (heelwalkers) |  | **0** |
| Mecoptera (scorpionflies) |  | **4** |
| Megaloptera |  | **8** |
| Neuroptera (net-winged insects) |  | **15** |
| Odonata (dragonflies & damselflies) |  | **34** |
| Orthoptera (grasshoppers. crickets & katydids) |  | **48** |
| Phasmatodea (walking sticks) |  | **22** |
| Plecoptera (stoneflies) |  | **23** |
| Psocodea (booklice. barklice & lice) |  | **19** |
| Raphidioptera (snakeflies) |  | **4** |
| Siphonaptera (fleas) |  | **3** |
| Strepsiptera (twisted-wing parasites) |  | **4** |
| Thysanoptera (thrips) |  | **15** |
| Trichoptera (caddisflies) |  | **73** |
| Zoraptera (angel insects) |  | **0** |
| Zygentoma |  | **3** |

Supplementary Table S3. Results of NCBI databases searches against WGS insecta and nucleotide collection with 11 satDNAs of *T. molitor*. The table includes the values for the hit with the highest identity and query coverage.

|  | **NCBI WGS insecta** | | | | **NCBI nucleotide collection** | | | |
| --- | --- | --- | --- | --- | --- | --- | --- | --- |
| **satDNA** | **species** | **order** | **Query cover (%)** | **Identity (%)** | **species** | **order** | **Query cover (%)** | **Identity (%)** |
| **TmSat1 (142 bp)** | *Cataglyphis bombycina* | Hymenoptera | 100 | 100 | *Tenebrio obscurus* | Coleoptera | 99 | 78.3 |
|  | *Cataglyphis mauritanica* | Hymenoptera | 100 | 100 |  |  |  |  |
|  | *Achroia grisella* | Lepidoptera | 100 | 100 |  |  |  |  |
|  | *Zophobas morio* | Coleoptera | 100 | 100 |  |  |  |  |
|  | *Solenopsis invicta* | Hymenoptera | 100 | 100 |  |  |  |  |
|  | *Cataglyphis hellenicus* | Hymenoptera | 100 | 99.30 |  |  |  |  |
|  | *Periplaneta americana* | Blattodea | 100 | 98.59 |  |  |  |  |
|  | *Cataglyphis cursor* | Hymenoptera | 100 | 97.89 |  |  |  |  |
|  | *Cataglyphis holgerseni* | Hymenoptera | 100 | 100 |  |  |  |  |
|  | *Cataglyphis italica* | Hymenoptera | 100 | 100 |  |  |  |  |
|  | *Cataglyphis velox* | Hymenoptera | 100 | 100 |  |  |  |  |
|  | *Cataglyphis albicans* | Hymenoptera | 100 | 99.12 |  |  |  |  |
| **TmSat2 (180 bp)** | *Cataglyphis albicans* | Hymenoptera | 100 | 100 |  |  |  |  |
|  | *Cataglyphis bombycina* | Hymenoptera | 100 | 96.58 |  |  |  |  |
| **TmSat3 (325 bp)** | *Phosphuga atrata* | Coleoptera | 28 | 77.6 | *Phosphuga atrata* | Coleoptera | 29 | 78.7 |
|  | *Drosophila melanica* | Diptera | 11 | 97.3 | *Aelia acuminata* | Hemiptera | 12 | 90.0 |
|  | *Drosophila micromelanica* | Diptera | 10 | 100 |  |  |  |  |
|  | *Cataglyphis albicans* | Hymenoptera | 100 | 95.44 |  |  |  |  |
| **TmSat4 (245 bp)** | *Podabrus alpinus* | Coleoptera | 36 | 76.6 | *Podabrus alpinus* | Coleoptera | 64 | 75.5 |
|  | *Cataglyphis albicans* | Hymenoptera | 100 | 99.52 | *Cantharis rustica* | Coleoptera | 25 | 90.0 |
|  | *Cataglyphis bombycina* | Hymenoptera | 89 | 97.46 | *Bemisia tabaci* | Hemiptera | 15 | 89.5 |
|  | *Hydrotaea cyrtoneurina* | Diptera | 25 | 91.11 | *Rhagonycha fulva* | Coleoptera | 17 | 88.1 |
|  | *Arthaldeus pascuellus* | Hemiptera | 22 | 84.91 | *Hydrotaea cyrtoneurina* | Diptera | 22 | 88 |
|  |  |  |  |  | *Phlebotomus argentipes* | Diptera | 26 | 79.37 |
|  |  |  |  |  | *Anaspis frontalis* | Coleoptera | 18 | 86.36 |
| **TmSat5 (364 bp)** | *Cataglyphis albicans* | Hymenoptera | 100 | 92.86 | *Miltochrista miniata* | Lepidoptera | 11 | 88.4 |
|  | *Prionychus ater* | Coleoptera | 53 | 86.84 | *Prionychus ater* | Coleoptera | 53 | 87.50 |
|  | *Heliconius aoede* | Lepidoptera | 18 | 83.08 |  |  |  |  |
| **TmSat6 (227 bp)** | *Achroia grisella* | Leptidopera | 100 | 100 | *Melinaea menophilus* | Lepidoptera | 17 | 92.7 |
|  | *Periplaneta americana* | Blattodea | 95 | 93.52 | *Synanthedon formicaeformis* | Lepidoptera | 22 | 82.7 |
|  | *Cataglyphis albicans* | Hymenoptera | 97 | 93.40 | *Chloroclysta siterata* | Lepidoptera | 19 | 84.4 |
|  |  |  |  |  | *Melinaea marsaeus rileyi* | Lepidoptera | 16 | 89.7 |
| **TmSat7 (189 bp)** | *Dinoponera quadriceps* | Hymenoptera | 100 | 100 |  |  |  |  |
|  | *Cataglyphis albicans* | Hymenoptera | 100 | 99.47 |  |  |  |  |
|  | *Achroia grisella* | Leptidopera | 100 | 98.94 |  |  |  |  |
|  | *Periplaneta americana* | Blattodea | 88 | 97.06 |  |  |  |  |
| **TmSat8 (735 bp)** | *Zophobas atratus* | Coleoptera | 18 | 82.35 | *Prionychus ater* | Coleoptera | 13 | 86.52 |
|  | *Tribolium madens* | Coleoptera | 16 | 75.6 | *Dendarus foraminosus* | Coleoptera | 13 | 81.91 |
|  | *Tribolium freemani* | Coleoptera | 13 | 73.9 |  |  |  |  |
|  | *Cataglyphis albicans* | Hymenoptera | 74 | 95.18 |  |  |  |  |
|  | *Zophobas morio* | Coleoptera | 16 | 79.82 |  |  |  |  |
|  | *Prionychus ater* | Coleoptera | 15 | 85.39 |  |  |  |  |
|  | *Blaps rhynchoptera* | Coleoptera | 12 | 77.42 |  |  |  |  |
| **TmSat9 (108 bp)** | *Cataglyphis albicans* | Hymenoptera | 100 | 93.33 |  |  |  |  |
| **TmSat10 (150 bp)** | *Cataglyphis albicans* | Hymenoptera | 100 | 88.24 | *Tribolium freemani* | Coleoptera | 54 | 76.8 |
|  | *Zophobas morio* | Coleoptera | 79 | 85.71 | *Vespula vulgaris* | Hymenoptera | 30 | 86.7 |
|  | *Tribolium confusum* | Coleoptera | 78 | 73.7 | *Thymelicus sylvestris* | Lepidoptera | 30 | 87.0 |
|  | *Tribolium freemani* | Coleoptera | 54 | 76.8 | *Dailognatha quadricollis* | Coleoptera | 96 | 72.37 |
|  | *Zophobas atratus* | Coleoptera | 57 | 85.71 | *Tribolium castaneum* | Coleoptera | 42 | 80.95 |
|  | *Asbolus verrucosus* | Coleoptera | 77 | 75.56 | *Tribolium freemani* | Coleoptera | 45 | 80.30 |
|  | *Blaps rhynchoptera* | Coleoptera | 61 | 81.82 | *Tribolium madens* | Coleoptera | 43 | 78.12 |
|  | *Gnatocerus cornotus* | Coleoptera | 77 | 73.83 | *Ligdia adustata* | *Lepidoptera* | 25 | 89.47 |
|  | *Latheticus oryzae* | Coleoptera | 52 | 79.49 |  |  |  |  |
|  | *Tribolium castaneum* | Coleoptera | 55 | 76.83 |  |  |  |  |
|  | *Cynaeus angustus* | Coleoptera | 49 | 78.08 |  |  |  |  |
| **TmSat11 (93 bp)** | No positive results |  |  |  |  |  |  |  |
